# Redescription of *Damastes leo* (Simon, 1903), comb. nov. (Araneae, Sparassidae) from Madagascar with first description of the male

**DOI:** 10.3897/zookeys.1282.192182

**Published:** 2026-06-17

**Authors:** Illia Uharov, Konrad Wiśniewski, Łukasz Trębicki, Peter Jäger

**Affiliations:** 1 Pomeranian University in Słupsk, Laboratory Support Group, Arciszewskiego 22a, 76-200 Słupsk, Poland University of Łódź, Faculty of Biology and Environmental Protection, Department of Invertebrate Zoology and Hydrobiology Łódź Poland; 2 Pomeranian University in Słupsk, Institute of Biology, Department of Zoology, Arciszewskiego 22a, 76-200 Słupsk, Poland Pomeranian University in Słupsk, Laboratory Support Group Słupsk Poland; 3 University of Łódź, Faculty of Biology and Environmental Protection, Department of Invertebrate Zoology and Hydrobiology, Banacha 12/16; 90-232 Łódź, Poland Pomeranian University in Słupsk, Institute of Biology, Department of Zoology Słupsk Poland; 4 Senckenberg Research Institute, Frankfurt am Main, Germany Senckenberg Research Institute Frankfurt am Main Germany

**Keywords:** Distribution, DNA barcoding, huntsman spider, rainforest, Ranomafana National Park

## Abstract

*Damastes leo* (Simon, 1903), **comb. nov**. (ex *Megaloremmius*) female is redescribed and the male is described for the first time. The species is endemic to Madagascar, and its range appears to be limited to remnants of the rainforest. The first COI record for the species is presented, and its distribution is analysed in detail using iNaturalist data.

## Introduction

There has been a recent increase in publications on the spiders of Madagascar. This trend encompasses not only the description of new species, but also the identification of numerous new genera spanning various spider groups over the past decade (Jocqué and Henrard 2015; Wood and Scharff 2018; Haddad 2021; Henrard et al. 2024; Kuntner et al. 2025; Pett et al. 2025). This trend highlights the scarcity of studies on Madagascar’s fauna and its distinctiveness. This also applies to the family Sparassidae, which is one of the most diverse families of spiders. Currently, this family comprises over 1,500 species worldwide (World Spider Catalog 2026), including 72 species in 13 genera recorded in Madagascar (Jäger 2020, 2021a, 2021b; Wood and Griswold 2022). Notably, 97% of these species are endemic to Madagascar. Recent studies on the Sparass­idae of Madagascar have described or revised highly distinct, endemic genera (Silva-Dávila 2005; Jäger 2020, 2021a, 2021b). In this study, we argue that there is one genus of Sparassidae too many in Madagascar. The first detailed study of a male of the monotypic genus *Megaloremmius* Simon, 1903 prompted its reclassification to the genus *Damastes* Simon, 1880.

Simon (1903) established *Megaloremmius* to classify the large, very hairy huntsman spider, *Megaloremmius leo* Simon, 1903. Simon differentiated it from the other Madagascan genus, *Damastes*, based on clypeal height. The common features of all *Damastes* include: both eye rows procurved, AME largest, embolus filiform and resting in a sheath-like conductor, and epigyne with lateral lobes touching along the median line (Jäger and Kunz 2005).

So far, only the female of *Damastes leo* comb. nov. has been officially recorded. There is just one additional paper by Jäger and Kunz (2005) relevant to the taxonomy of this genus, in which the female genitalia are depicted. Aside from these contributions, the genus *Megaloremmius* remains poorly understood. However, a substantial number of photographs are available online, as these spiders are conspicuous, distinctive, and spectacular.

Thanks to the recent discovery of the species in Ranomafana National Park, especially the sampling of the male sex, we can redescribe the species and revise its taxonomic position. We also provide the first barcode (cytochrome c oxidase subunit I, COI) sequences for the identified species of *Damastes*; only a few sequences were available in GenBank, all from specimens identified to genus level only. Barcoding (Hebert et al. 2003) is a powerful tool in biodiversity studies, as it, for instance, supports sex-matching (Korai and Jäger 2025; Zhang et al. 2025). We used this method to support morphological data and to group extant COI sequences of *Damastes*.

## Materials and methods

### Collecting and illustrating

The specimens were collected in November 2024 in Ranomafana National Park. A female and a male of this species were sampled by hand at night and preserved in 70% ethanol. The male illustration was prepared on an iPad using photographs taken under a stereomicroscope with focus stacking and a camera lucida attached to a stereomicroscope. Setae and spines are omitted from the illustrations. Female copulatory organs were dissected, cleared in 96% lactic acid and drawn using a camera lucida attachment on a stereomicroscope. Habitus photos in ethanol were taken using a Canon EOS R5 with a Canon ringlite. All measurements are in millimetres. The leg formula and leg spination pattern follow Jäger (2001). As in Jäger and Kunz (2005: 88), slit sensilla near the epigyne are depicted and considered taxonomically relevant characters.

Additionally, we compare our species with *Damastes validus* (Blackwall, 1877), which we consider most similar to *D. leo* comb. nov. For this reason, we present new figures for the former species, based on specimens from museum collections. The specimens used for comparison has the following material data:

Seychelles • 1♀, 1 juv.; Silhouette; Brauer leg.; ZMB, PJ 864 • 1♂; MNHN, 1603. Fig. 5A–G

All specimens were checked for scars, as potential traces of mating bites as described by Jäger and Krehenwinkel (2015), Jäger (2021a, 2023), and Eudeline and Jäger (2023).

### Genetic data

Genomic DNA was extracted using a magnetic bead-based protocol specifically adapted for degraded and challenging museum specimens (Tin et al. 2014). The standard procedure was modified in two respects: (i) guanidine isothiocyanate in the lysis buffer was replaced with guanidine thiocyanate, and (ii) magnetic beads supplied by G-Biosciences (St. Louis, MO, USA) were substituted with Qiagen MagAttract Suspension G beads from the MagAttract HMW DNA Kit (Qiagen GmbH, Hilden, Germany). A single dissected leg was incubated in lysis buffer overnight at 56 °C.

The standard COI DNA barcoding fragment was amplified using PCR, after which amplicons were quality-checked and subjected to Sanger sequencing. PCR cycling conditions and post-PCR purification were performed according to the protocol outlined by Querner et al. (2022). Purified products were sequenced in the forward direction using the Sanger method, with sequencing services provided by Macrogen Europe BV (Amsterdam, The Netherlands).

One of the two Sanger-generated amplicons yielded a contaminated sequence. To overcome this issue, next-generation sequencing based on Oxford Nanopore Technologies (ONT) was employed, following the framework proposed by Srivathsan et al. (2021, 2024). PCR amplification, library preparation, sequencing, and downstream read processing were conducted according to the protocol described by Bienias et al. (2025).

Sequence assembly and alignment were conducted in Geneious v. 10.2.6 (Biomatters Ltd; Kearse et al. 2012), with multiple sequence alignment performed using MUSCLE (Edgar 2004) under default parameters. The resulting alignments were subsequently examined manually; primer sequences were trimmed, and all sequences were checked for the presence of stop codons, frameshifts, and ambiguous nucleotide calls. For Sanger-generated sequences produced by Macrogen, chromatograms were additionally inspected for double peaks and other signs of sequence ambiguity using the same software environment.

Sequence identification was carried out through similarity-based searches against the GenBank database using BLAST and by comparison with reference barcode records in the BOLD Systems database.

To confirm conspecificity of males and females, COI-based species delimitation was performed using sequences generated in this study, together with previously published and publicly available COI sequences of the genus *Damastes* (Table 1).

**Table 1.** Sequences included in analyses. The sequences originate from public databases and from spiders collected by us. The specimens available in the databases were not identified to the species level.

Accession number (GenBank)	Process ID (BOLD)	Identification	References
OK646491	GBMNE88745-22	*Damastes* sp.	Gorneau et al. 2022
OK646492	GBMNE88746-22	*Damastes* sp.	Gorneau et al. 2022
OK646493	GBMNE88747-22	*Damastes* sp.	Gorneau et al. 2022
OK646494	GBMNE88748-22	*Damastes* sp.	Gorneau et al. 2022
OK646495	GBMNE88749-22	*Damastes* sp.	Gorneau et al. 2022
OK646496	GBMNE88750-22	*Damastes* sp.	Gorneau et al. 2022
GQ855822	GBCH5137-10	*Damastes* sp. SB-2009	Unpublished
HM575918	GACS542-19	*Damastes* sp.	Crews and Gillespie 2010
PZ179808	AFARA115-25	*Damastes leo* comb. nov.	This study
PZ179809	AFARA116-25	*Damastes leo* comb. nov.	This study
—	SPIZA996-21	*Olios* sp.	National Collection of Arachnida, South Africa

Molecular operational taxonomic units (OTUs) were delineated on the Barcode of Life Data Systems (BOLD) platform using the Refined Single Linkage (RESL) algorithm. High-quality sequences were assigned to Barcode Index Numbers (BINs) following the standard BOLD workflow (Ratnasingham and Hebert 2013). A neighbour-joining (NJ) distance tree (Saitou and Nei 1987) was constructed based on Kimura 2-parameter (K2P) distances (Kimura 1980), with nodal support assessed using 500 bootstrap replicates (Felsenstein 1985). Estimates of intraspecific genetic distances were calculated using the K2P model in MEGA12 (Kumar et al. 2024).

All sequences generated in this study have been deposited in the BOLD Systems database (Ratnasingham and Hebert 2007) and are publicly accessible under doi: https://doi.org/10.5883/ds-damasleo. The same sequences have also been submitted to GenBank: PZ179808–PZ179809.

### Distribution analysis

The distribution map of *Damastes leo* comb. nov. was prepared using publicly available data from iNaturalist (Suppl. material 1). The presence of a similar species in Madagascar cannot be totally ruled out, but it seems unlikely that such a massive and conspicuous species have been overlooked for centuries. However, our interpretation of the records from iNaturalist may be taken with some caution. The map was created in SimpleMappr (Shorthouse 2010) and subsequently modified in PhotoScape X.

### Abbreviations

**ALE**—anterior lateral eyes, **AME**—anterior median eyes, **AW**—anterior width of prosoma, **OL**—opisthosoma length, **OW**—opisthosoma width, **PL**—prosoma length, **PLE**—posterior lateral eyes, **PME**—posterior median eyes, **PW**—prosoma width, **I–IV**—legs I–IV.

**MNHN** Muséum national d’Histoire naturelle, Paris, France (Kaïna Privet);

**SMF** Senckenberg Research Institute, Arachnology, Frankfurt am Main, Germany (Peter Jäger);

**ZMB** Museum für Naturkunde – Leibniz Institute for Evolution and Biodiversity Science, Berlin, Germany (Jason Dunlop).

## Results

### Taxonomy

**Sparassidae Bertkau, 1872**

***Damastes* Simon, 1880**

#### Damastes leo

Taxon classificationAnimaliaAraneaeSparassidae

(Simon, 1903) comb. nov.

44030826-6A38-560A-A4D0-CCD7AE73BECD

**Figure 1.**
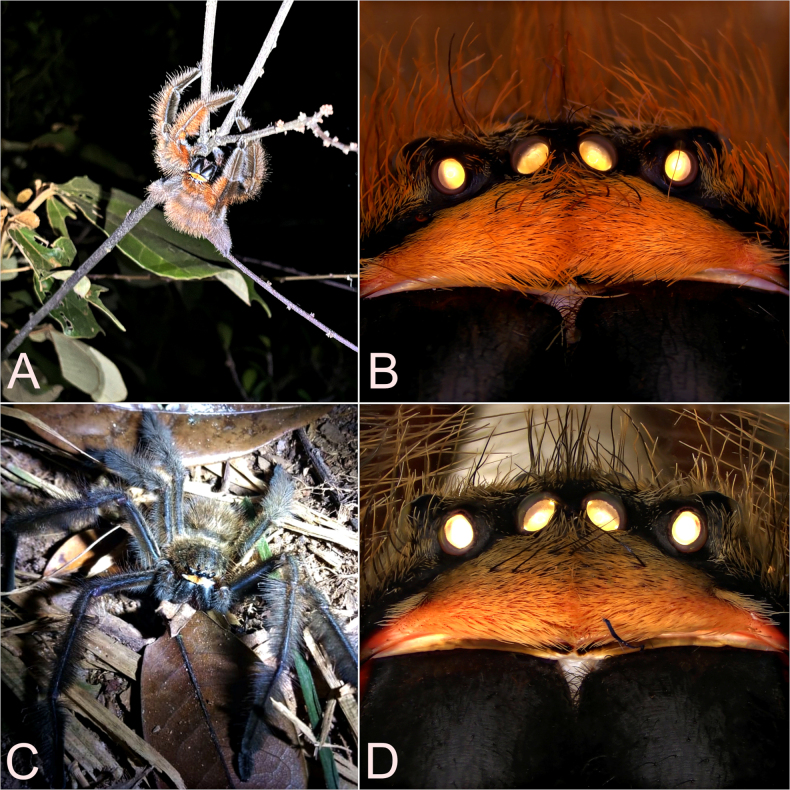
*Damastes leo* comb. nov. in the Ranomafana National Park. **A**. Female in the treetops over the Namorona River, ca 5–10 m above ground level (photo by Michał Furgoł); **B, D**. Clypeus, frontal (B female, D male); **C**. Male in rainforest litter (photo by George Toussaint).

**Figure 2.**
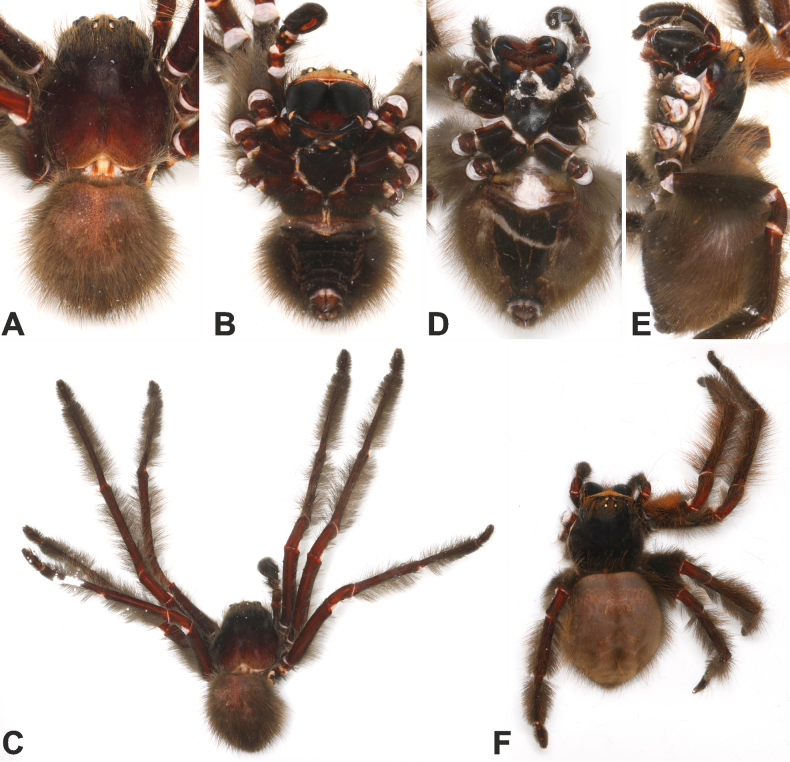
Habitus of *Damastes leo* comb. nov. **A–C**. Male; **D–F**. Female. **A, C, F**. Dorsal view; **B, D**. Ventral view; **E**. Lateral view.

**Figure 3.**
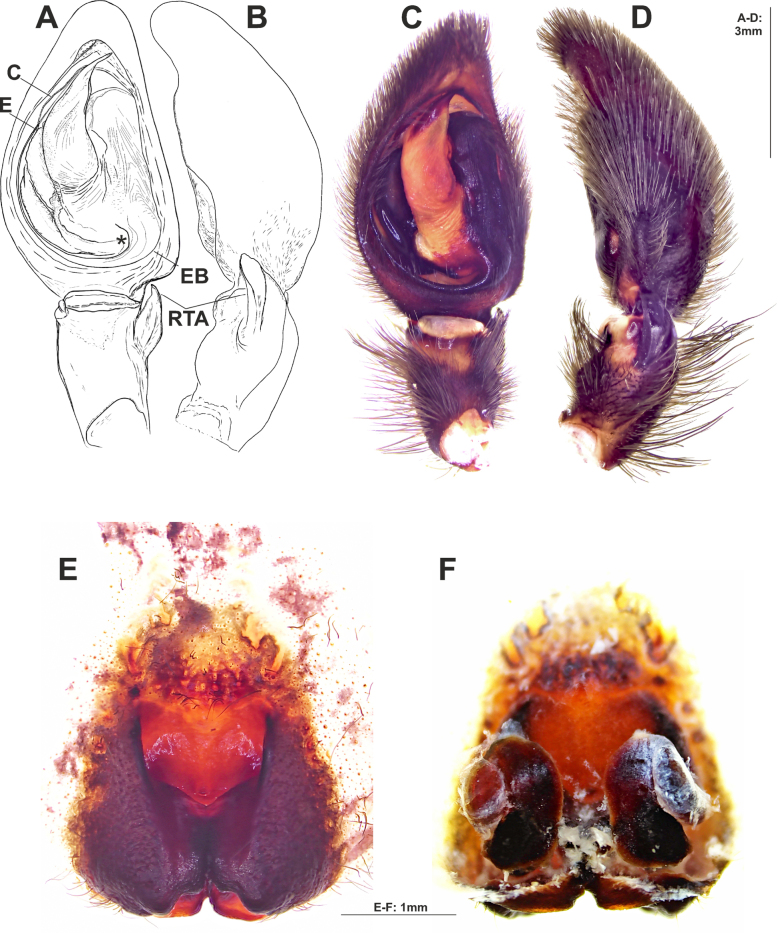
*Damastes leo* comb. nov. **A–D**. Male; **E, F**. Female. **A, C**. Left pedipalp, ventral view; **B, D**. Same, retrolateral view; **E**. Epigyne, ventral view; **F**. Vulva, dorsal view. Abbreviations: C – conductor, E – embolus, EB – embolus base, RTA – retrolateral apophysis. Asterisk indicates U-shaped structure at embolus base.

**Figures 4.**
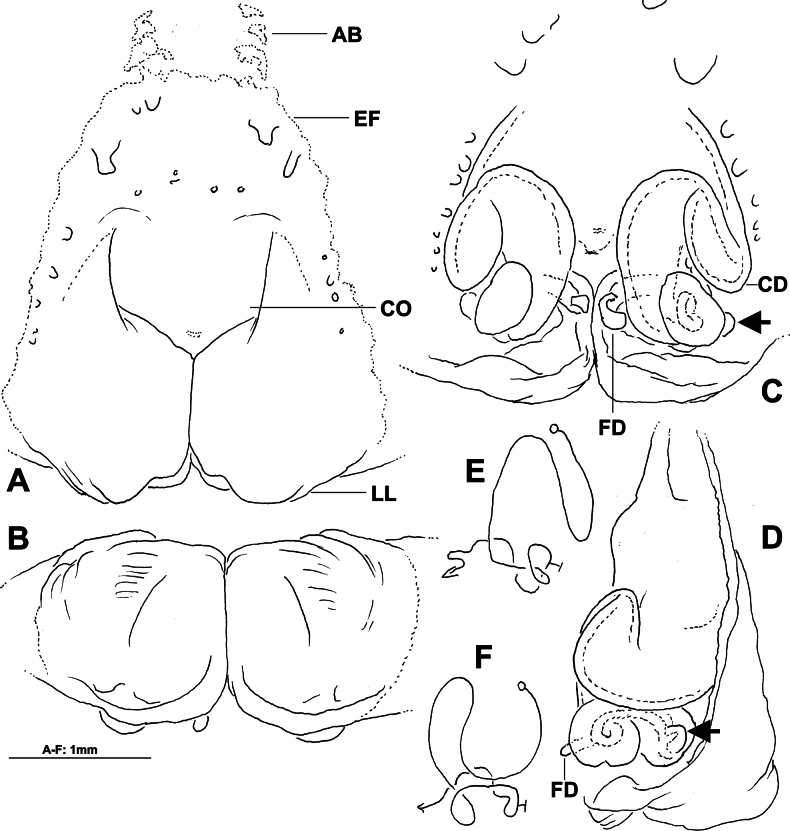
*Damastes leo* comb. nov., female. **A**. Epigyne, ventral view; **B**. Epigyne, posterior view; **C**. Vulva, dorsal view; **D**. Vulva, lateral view; **E**. Schematic course of internal duct system, dorsal view; **F**. Same, lateral view. Abbreviations: AB – anterior band, CD – copulatory duct, CO – copulatory opening, EF – epigynal field, FD – fertilization duct, LL – lateral lobe. Arrows indicating glandular appendages.

Figs 1A–D, 2A–F, 3A–F, 4A–F

Megaloremmius leo Simon, 1903: 1025 (Description of female; holotype female from Antongil [ca 15°26'7.7"S, 49°43'52.8"E, 15 m a.s.l.], Madagascar, MNHN 1624-21398; examined). Jäger and Kunz 2005: 167, figs 103–108 (illustration of female).

##### Material examined.

Madagascar • 1 ♂; Ranomafana National Park, Valohoaka site; 21°19'12.0"S, 47°25'12.0"E (ca 1 km accuracy); ca 1100 m a.s.l.; 27 Nov. 2024; George Toussaint leg.; night, in litter, hand collected; BOLD/GenBank: [AFARA115-25]; SMF • 1 ♀, Ranomafana National Park, by Namorona River; 21°15'29.7"S, 47°25'17.4"E; 920 m a.s.l.; 25 Nov. 2024; Konrad Wiśniewski leg.; night, hand collected, in the tree top, ca 10 m above ground level, collected from the bridge over Namorona; BOLD/GenBank: [AFARA116-25]; SMF.

##### Diagnosis.

**Figures 5.**
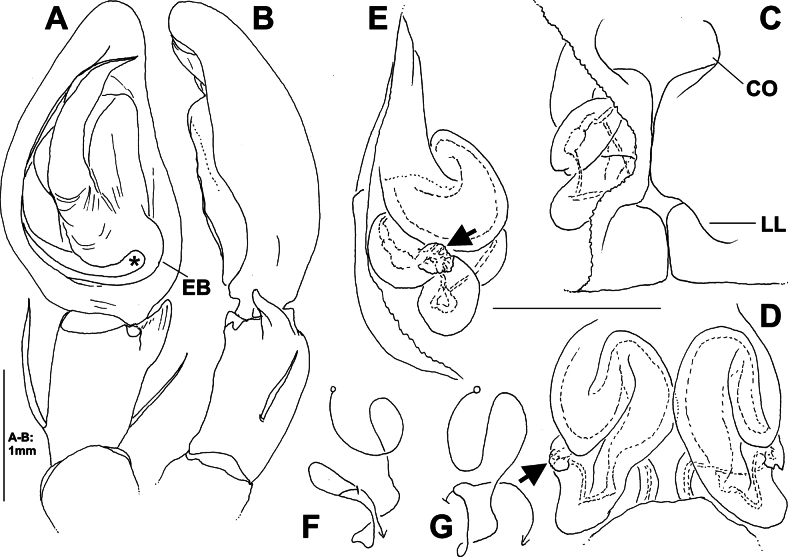
Copulatory organs of *Damastes validus*. **A**. Male, left pedipalp, ventral view; **B**. Same, retrolateral view; **C–G**. Female; **C**. Epigyne, ventral view, with some internal structures visible (epigyne partly damaged); **D**. Vulva, ventral view; **E**. Vulva, left lateral view; **F**. Schematic course of internal duct system, left lateral view; **G**. Same, dorsal view. Abbreviations: CO – copulatory opening, EB – embolus base, LL – lateral lobe. Asterisk indicating U-shaped structure at embolus base, arrows indicating glandular appendages.

The species is highly characteristic due to its large size, dense and long setae, and the clypeus covered densely with orange-red setae. Compared with all other *Damastes*, it is larger and more robust, with a more convex carapace (Fig. 2E). The male pedipalp of *D. leo* (Fig. 3A–D) is similar to that of *Damastes validus* (Fig. 5A, B), sharing the general arrangement of the pedipalpal elements, such as the embolus and conductor, with the embolus base bulging retrolaterally, but can be distinguished by: 1. Conductor wider at its base, narrowing strongly towards tip (generally narrower and of more equal width in *D. validus*); 2. Embolus base forming prolaterally a shallow U-shaped margin (forming a distinct elongated U-shaped margin in *D. validus*). The female copulatory organ of *D. leo* (Figs 3E, 3F, 4A–F) resemble that of *D. validus* (Fig. 5C–G) in having a similar structure of the internal duct system, i.e. the copulatory ducts firstly running posterior from the antero-laterally situated openings, then running in a sigmoid double turn, after that running as a broad duct along the median line to the posterior part with some postero-lateral coils before entering the fertilization ducts dorso-posteriorly, but can be distinguished by: 1. Distance between ducts clearly separated (situated close to each other in *D. validus*). 2. Lateral lobes protruding beyond the epigastric furrow posteriorly (not so in *D. validus*). 3. Glandular appendages ventrad (dorsad in *D. validus*).

##### Description of male.

Spider large, total length 29.2; carapace longer than wide, convex; brownish black in living specimens (Fig. 1C); brown to dark brown in preserved specimens (Fig. 2A–C); clypeus relatively high for *Damastes*, i.e., two times the diameter of AME, yellowish brown (Figs 1D, 2B). Legs long, slender, with long, red, brown to bluish-iridescent setae (Fig. 1C). Pedipalp (Fig. 3A–D) as in diagnosis. RTA arising subdistally from tibia, wide at base, tapering and slightly curved to ventral side (retrolateral view). Cymbium longer than tibia, bluntly rounded apically. Conductor arising centrally from tegulum, broad at base, tapering, its tip retrolatero-distad, joining embolus at 10:30. Spermophor not visible due to sclerotization. Embolus arising point at 4:30, base bulging retrolaterally, semicircular.

PL 14.5, PW 12.8, AW 8.7, OL 14.7, OW 11.1; Eyes: AME 0.90, ALE 0.71, PME 0.70, PLE 0.69; AME–AME 0.43, AME–ALE 0.90, PME–PME 0.99, PME–PLE 171, AME–PME 0.60, ALE–PLE 0.70, clypeus height at AME 2.03, clypeus height at ALE 1.65. Chelicerae with 3/2 promarginal, 5 retromarginal teeth, without denticles.

Spination: pedipalp: 100, 000, 000; legs: Femur I 203(302), II 313(4), III 312, IV 300; Patella 000; Tibia I 0(2)006, II 2026, III–IV 2006; Metatarsus I–II 0003, III 1004, IV 1005.

Measurements of pedipalp and legs: pedipalp 17.6 (5.9, 2.3, 3.3, -, 6.1), I 60.2 (16.2, 7.1, 15.8, 16.9, 4.3), II 62.8 (17.1, 7.2, 16.9, 17.3, 4.3), III 47.3 (14.2, 5.8, 12.3, 11.4, 3.6), IV 47.0 (14.1, 5.4, 11.9, 11.6, 4.0). Leg formula 2134.

Scars: left tibia IV with 1 round and 1 irregular scar dorsally in distal half.

##### Description of female.

Large spider, total length 35.2, carapace longer than wide, convex, dark brown, clypeus yellow-orange, muted orange in preserved specimens. Legs long, more robust than in male, legs and opisthosoma with dense, reddish-brown setae (Fig. 1A). Epigyne and internal copulatory organs as in diagnosis and Figs 3E, 3F, 4A–F(compare also: Jäger and Kunz 2005: figs 103–105). Epigynal field trapezoid, distinctly longer than wide, anterior bands patchy, indistinct. Slit sensilla absent. Copulatory openings situated antero-laterally.

PL 14.7, PW 13.1, AW 9.5, OL 20.5, OW 16.7; Eyes: AME 0.84, ALE 0.62, PME 0.70, PLE 0.62; AME–AME 0.43, AME–ALE 0.95, PME–PME 1.13, PME–PLE 1.89, AME–PME 0.59, ALE–PLE 0.75, clypeus height at AME 1.61, clypeus height at ALE 1.32. Chelicerae with 3/2 promarginal, 4/3 retromarginal teeth, without denticles.

Spination: pedipalp: 100, 000, 1000, 2(1)010; legs: FE I 113, II 313, III 312(3), IV 311; PA 000; TI I 0006, II–IV 1006; MT I 0003, II 0004, III 1004, IV 1005.

Measurements of pedipalp and legs: pedipalp 16.1 (5.1, 2.8, 3.1, —, 5.1), I 47.8 (13.2, 6.5, 12.4, 12.2, 3.5), II 50.0 (14.1, 6.7, 13.0, 12.6, 3.6), III 40.2 (12.5, 5.5, 10.1, 8.6, 3.5), IV 49.1 (11.9, 5.3, 9.8, 8.6, 3.5). Leg formula 2413.

##### Remarks.

We found intraspecific variation in the shape of the posterior lateral lobes in the epigyne of the present female and the holotype illustrated by Jäger and Kunz (2005: fig. 103): the posterior humps of the lateral lobes in the holotype are much longer and more defined than those in our specimen (Figs 3E, 4A).

We propose the English name “Malagasy lion spider” for the species, which refers to its Latin name and appearance.

##### Distribution.

**Figure 6.**
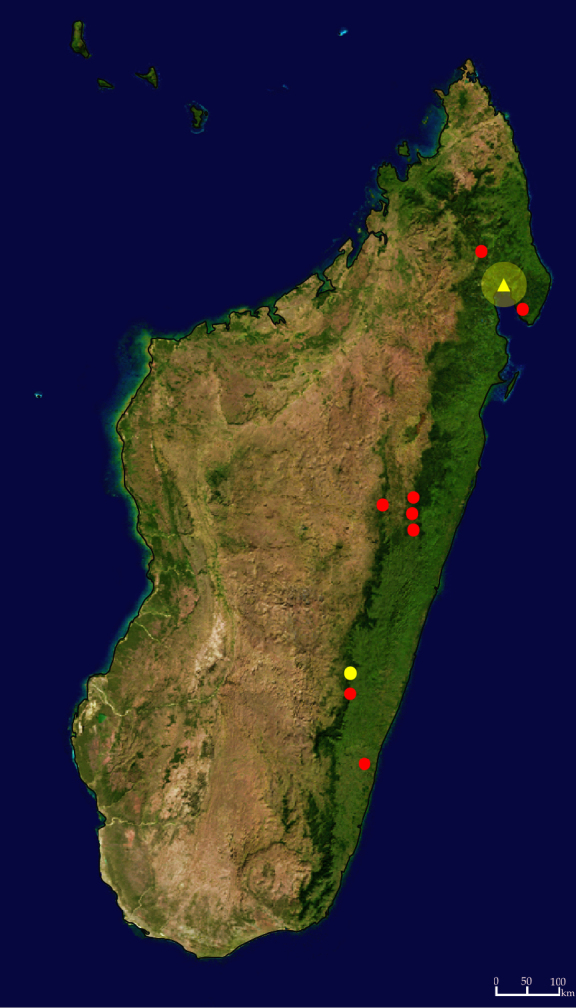
The known distribution of *Damastes leo* comb. nov. based on data from iNaturalist (red dots), including the type locality (yellow triangle) and the collection presented in this article (yellow dot). The dark green colour indicates the current extent of Madagascar’s rainforests.

The range of this species appears to be restricted to the remnants of the rainforest in eastern Madagascar (Fig. 6, Suppl. material 1). Most records come from the north-east (Alaotra-Mangoro), central east (environs of Andasibe, Moramanga, and Mantadia National Park) and more to the south, in the district of Fiaranantsoa (e.g. Ranomafana National Park).

##### Molecular data.

**Figure 7.**
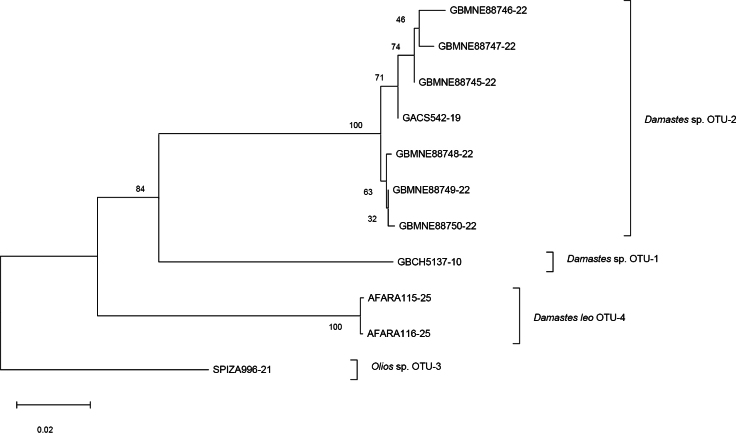
Neighbour-joining tree of the COI sequences representing the *Damastes* species and the *Olios* representative as an outgroup. Numbers near nodes show bootstrap values.

The two sequences obtained from specimens originating from Ranomafana National Park were identical (Table 2). No other publicly available sequences are available in the repositories. We observed considerable similarity in COI sequences with those of other representatives of the genus *Damastes* (Table 2; Fig. 7).

**Table 2.** OTUs from Cluster Sequence (RESL) Analysis.

OTU	Process ID	Mean	Max	Taxon	BIN	Count	NN Dist
OTU-1	GBCH5137-10	0	0	*Damastes* sp. SB-2009	BOLD:AAN1412	1	11.0732
OTU-2	GACS542-19	0.8414	2.0446	*Damastes* sp.		7	11.0732
OTU-2	GBMNE88745-22	0.8414	2.0446	*Damastes* sp.		7	11.0732
OTU-2	GBMNE88746-22	0.8414	2.0446	*Damastes* sp.		7	11.0732
OTU-2	GBMNE88747-22	0.8414	2.0446	*Damastes* sp.		7	11.0732
OTU-2	GBMNE88748-22	0.8414	2.0446	*Damastes* sp.	BOLD:AEW8282	7	11.0732
OTU-2	GBMNE88749-22	0.8414	2.0446	*Damastes* sp.	BOLD:AEW8282	7	11.0732
OTU-2	GBMNE88750-22	0.8414	2.0446	*Damastes* sp.	BOLD:AEW8282	7	11.0732
OTU-3	SPIZA996-21	0	0	*Olios* sp.	BOLD:AEO0918	1	13.7037
OTU-4	AFARA115-25	0.1634	0.1634	*Damastes leo*		2	12.9808
OTU-4	AFARA116-25	0.1634	0.1634	*Damastes leo*		2	12.9808

The final alignment used for species delimitation comprised 658 nucleotide positions (nps) of COI gene sequences representing 11 specimens (Table 2). All nucleotide sequences were successfully translated into amino acid sequences without stop codons. Of the 658 nucleotide positions, 163 were variable, and the transition-to-transversion ratio (R) was 1.04.

The NJ analysis clustered the COI sequences of *Damastes* into maximally supported clades (Table 3; Fig. 7), grouping male and female specimens of *Damastes leo* comb. nov. together. Genetic divergence among COIOTUs ranged from 14.5% to 17.25%.

**Table 3.** Estimates of Evolutionary Divergence over Sequence Pairs between OTU groups.

	*Olios* sp. OTU-3	*Damastes leo* OTU-4	*Damastes* sp. OTU-1	*Damastes* sp. OTU-2
*Olios* sp. OTU-3				
***Damastes leo* OTU-4**	15.60%			
*Damastes* sp. OTU-1	17.25%	14.50%		
*Damastes* sp. OTU-2	16.51%	15.68%	13.02%	

## Discussion

### Taxonomic status

*Damastes leo* comb. nov. was originally assigned to a monotypic genus, *Megalo­remmius*, based on its distinctive appearance (Simon 1903). However, a thorough morphological analysis of the first discovered male indicates that placement in a separate genus is not justified, as most of its features, especially the structure of the genitalia, are similar to those of other *Damastes* species. The position and shape of the embolus, the shape of the conductor, the general structure of the RTA, and the number of cheliceral teeth are all typical of *Damastes*. Therefore, maintaining the monotypic genus is unnecessary. The diversity of *Damastes* might be considerable and larger than previously thought. The genus requires a complete revision, with several undescribed or unidentified species (Soutinho et al. 2018; Fulgence et al. 2021; Gorneau et al. 2023). The phylogenetic placement of the genus *Damastes* within Sparassidae remains unresolved; although it has been included in several molecular phylogenetic studies (e.g. Agnarsson and Rayor 2013; Moradmand et al. 2014; Gorneau et al. 2022, 2023) and consistently recovered as monophyletic, its affinities remain weakly supported and inconsistent, being variably placed near Palystinae or outside major subfamilial clades, precluding confident assignment to any currently recognised subfamily. Further molecular data are required to clarify its phylogenetic relationships.

### Distribution and conservation issues

*Damastes leo* comb. nov. is highly distinctive; however, the only published record to date is from Simon (1903: Antongila Bay). Thanks to its distinctive appearance, this spider has been well documented on iNaturalist. However, the data from this source are probably incomplete and primarily reflect popular tourist and naturalist destinations (e.g. Andasibe and Ranomafana). Specimens observed in other localities do not differ significantly in overall appearance. Nevertheless, the possibility of sibling species occurrence cannot be completely excluded.

The species is exclusively found in the rainforests of eastern Madagascar and most images were taken in forest habitats. It is likely to be a good indicator species for rainforests, which are currently under severe threat in Madagascar (Vieilledent et al. 2018). However, this must be confirmed by further observations. Conserving rainforest areas and their primary characteristics is essential for maintaining high biodiversity among numerous animal groups (Rajaonarimalala et al. 2024).

Most observations of *D. leo* comb. nov. originate from easily accessible locations, as spiders were photographed near or at ground level. However, the species is active across a variety of microhabitats, including tree crowns, as our observation of a female clearly demonstrates. This habitat is generally difficult for scientists to access and supports significant spider diversity in rainforests, although data on it are limited (Russell-Smith and Stork 1994).

### Molecular data

We presented the first barcode sequences for this species, as well as the first sequences for other members of the genus *Damastes* that remain unidentified. COI sequences have proven highly useful for sex matching (Trębicki et al. 2021) and community studies (Kennedy et al. 2020). While there was little doubt that the female and male we observed belonged to the same species, there may still be hidden diversity within *D. leo* populations, and it would be worthwhile to check all specimens for cryptic species.

The COI divergence obtained for *Damastes* from databases indicate comparatively high interspecific divergence (Zhang et al. 2025), ranging from 13 to 17%. This supports the conclusion that these are distinct species. However, subsequent work must include more species from this potentially large genus, as well as additional genetic markers (Gajski et al. 2024), to support diversity analysis and phylogenetic inference.

## Supplementary Material

XML Treatment for Damastes leo

## Additional information

### Artificial Intelligence (AI) use

The authors accept full responsibility for the content of the manuscript, including the disclosure of any use of AI.

No AI tools were used in the preparation of this manuscript.

### Author ORCIDs

I. Uharov https://orcid.org/0009-0007-9840-9048

K. Wiśniewski https://orcid.org/0000-0002-6780-3292

Ł. Trębicki https://orcid.org/0000-0002-6384-226X

P. Jäger https://orcid.org/0000-0003-1563-0147

### Data availability

All of the data that support the findings of this study are available in the main text or Supplementary Information.

## Supplementary materials

Supplementary material 1 Data on the distribution of *Damastes leo* from iNaturalist Data type docx Explanation note **table SS1**: Data on the distribution of *Damastes leo* from iNaturalist. Coordinates and geographical regions are named and standardized, as well as the sex of the spotted spider. The last check was carried out on 1 October 2025. m – male, f – female. https://binary.pensoft.net/file/1682665This dataset is made available under the Open Database License (http://opendatacommons.org/licenses/odbl/1.0/). The Open Database License (ODbL) is a license agreement intended to allow users to freely share, modify, and use this Dataset while maintaining this same freedom for others, provided that the original source and author(s) are credited. Illia Uharov, Konrad Wiśniewski, Łukasz Trębicki, Peter Jäger

## References

[B1] Agnarsson I, Rayor LS (2013) A molecular phylogeny of the Australian huntsman spiders (Sparassidae, Deleninae): implications for taxonomy and social behaviour. Molecular Phylogenetics and Evolution 69(3): 895–905. 10.1016/j.ympev.2013.06.015 23831456

[B2] Bertkau P (1872) Über die Respirationsorgane der Araneen. Archiv für Naturgeschichte 38: 208–233.

[B3] Blackwall J (1877) A list of spiders captured in the Seychelles Islands, by Professor E. Perceval Wright, M. D., F. L. S.; with descriptions of species supposed to be new to arachnologists. Proceedings of the Royal Irish Academy (2) 3(1): 1–22.

[B4] Bienias J, Voigtländer K, Trębicki Ł, Kościelniak J, Grabowski M (2025) Overlooked expansion? The case of the millipede *Polydesmus angustus* Latzel, 1884 in Poland. BioInvasions Record 14(1): 31–45. 10.3391/bir.2025.14.1.04

[B5] Crews SC, Gillespie RG (2010) Molecular systematics of *Selenops* spiders (Araneae: Selenopidae) from North and Central America: implications for Caribbean biogeography. Biological Journal of the Linnean Society 101(2): 288–322. 10.1111/j.1095-8312.2010.01494.x

[B6] Edgar RC (2004) MUSCLE: multiple sequence alignment with high accuracy and high throughput. Nucleic Acids Research 32: 1792–1797. 10.1093/nar/gkh340 PMC390337 15034147

[B7] Eudeline R, Jäger P (2023) First field observation of a mating bite of *Thunberga* sp. Newsletter of the British Arachnological Society 158: 13–14.

[B8] Felsenstein J (1985) Confidence limits on phylogenies: an approach using the bootstrap. Evolution 39(4): 783–791. 10.1111/j.1558-5646.1985.tb00420.x 28561359

[B9] Fulgence TR, Martin DA, Kreft H, Ratsoavina FM, Andrianarimisa A (2021) Spider traps amphibian in northeastern Madagascar. Ecology and Evolution 11: 682–687. 10.1002/ece3.7102 PMC7820146 33520157

[B10] Gajski D, Wolff JO, Melcher A, Weber S, Prost S, Krehenwinkel H, Kennedy SR (2024) Facilitating taxonomy and phylogenetics: an informative and cost-effective protocol integrating long amplicon PCRs and third-generation sequencing. Molecular Phylogenetics and Evolution 192: 107988. 10.1016/j.ympev.2023.107988 38072140

[B11] Gorneau JA, Rheims CA, Moreau CS, Rayor LS (2022) Huntsman spider phylogeny informs evolution of life history, egg sacs, and morphology. Molecular Phylogenetics and Evolution 174: 107530. 10.1016/j.ympev.2022.107530 35636670

[B12] Gorneau JA, Rayor LS, Rheims CA, Moreau CS (2023) Molecular, morphological, and life history data to support research of huntsman spiders (Araneae: Sparassidae). Data in Brief 46: 108885. 10.1016/j.dib.2023.108885 PMC9868322 36699733

[B13] Haddad CR (2021) *Griswoldella* gen. nov., a new castianeirine spider genus from Madagascar (Araneae: Corinnidae). Arachnology 18: 859–866. 10.13156/arac.2021.18.8.859

[B14] Hebert PDN, Cywinska A, Ball SL, de Waard JR (2003) Biological identifications through DNA barcodes. Proceedings of the Royal Society B: Biological Sciences 270: 313–321. 10.1098/rspb.2002.2218 PMC1691236 12614582

[B15] Henrard A, Griswold C, Jocqué R (2024) On new genera and species of crack-leg spiders (Araneae, Udubidae) from Madagascar. European Journal of Taxonomy 966: 1–80. 10.5852/ejt.2024.966.2697

[B16] Jäger P (2001) Diversität der Riesenkrabbenspinnen im Himalaya. Über eine Radiation zweier Gattungen in den Schneetropen (Araneae: Sparassidae: Heteropodinae). Courier Forschungsinstitut Senckenberg 232: 1–136.

[B17] Jäger P (2020) *Thunberga* gen. nov., a new genus of huntsman spiders from Madagascar (Araneae: Sparassidae: Heteropodinae). Zootaxa 4790(2): 245–260. 10.11646/zootaxa.4790.2.3 33055840

[B18] Jäger P (2021a) Enigmatic “love bites” and an indirect mating plug: revision of *Thunberga* Jäger, 2020 with description of new species (Araneae: Sparassidae: Heteropodinae). Arachnology 18(7): 718–765. 10.13156/arac.2020.18.7.718

[B19] Jäger P (2021b) Two new enigmatic genera of huntsman spiders from Madagascar (Araneae: Sparassidae). Zootaxa 4984(1): 335–346. 10.11646/zootaxa.4984.1.24 34186678

[B20] Jäger P (2023) Revision of the huntsman spider genus *Micrommata* Latreille, 1804 (Sparassidae: Sparassinae). Zootaxa 5352(1): 1–45. 10.11646/zootaxa.5352.1.1 38221461

[B21] Jäger P, Krehenwinkel H (2015) *May* gen. nov. (Araneae: Sparassidae): a unique lineage from southern Africa supported by morphological and molecular features. African Invertebrates 56: 365–392. 10.5733/afin.056.0209

[B22] Jäger P, Kunz D (2005) An illustrated key to genera of African huntsman spiders (Arachnida, Araneae, Sparassidae). Senckenbergiana Biologica 85(2): 163–213.

[B23] Jocqué R, Henrard A (2015) The new spider genus *Palindroma*, featuring a novel synapomorphy for the Zodariidae (Araneae). European Journal of Taxonomy 152: 1–33. 10.5852/ejt.2015.152

[B24] Kearse M, Moir R, Wilson A, Stones-Havas S, Cheung M, Sturrock S, Buxton S, Cooper A, Markowitz S, Duran C, Thierer T, Ashton B, Meintjes P, Drummond A (2012) Geneious Basic: an integrated and extendable desktop software platform for the organization and analysis of sequence data. Bioinformatics 28: 1647–1649. 10.1093/bioinformatics/bts199 PMC3371832 22543367

[B25] Kennedy SR, Prost S, Overcast I, Rominger AJ, Gillespie RG, Krehenwinkel H (2020) High-throughput sequencing for community analysis: the promise of DNA barcoding to uncover diversity, relatedness, abundances and interactions in spider communities. Development Genes and Evolution 230: 185–201. 10.1007/s00427-020-00652-x PMC7127999 32040713

[B26] Kimura M (1980) A simple method for estimating evolutionary rate of base substitutions through comparative studies of nucleotide sequences. Journal of Molecular Evolution 16: 111–120. 10.1007/BF01731581 7463489

[B27] Korai SK, Jäger P (2025) First molecular phylogeny of species of the genus *Heteropoda* (Sparassidae: Heteropodinae): implications for taxonomy. Insect Systematics and Diversity 9(4): 4. 10.1093/isd/ixaf010

[B28] Kumar S, Stecher G, Suleski M, Sanderford M, Sharma S, Tamura K (2024) MEGA12: Molecular Evolutionary Genetics Analysis version 12 for adaptive and green computing. Molecular Biology and Evolution 41: msae263. 10.1093/molbev/msae263 PMC11683415 39708372

[B29] Kuntner M, Yu K-P, Bedjanič M, Gregorič M, Turk E, Čandek K, Coddington JA, Agnarsson I, Starrett J, Bond JE (2025) *Osmooka*, a new spider genus from Madagascar: a surprising relative of the Australian fauna (Araneae: Paraplectanoididae). Insect Systematics and Diversity 9: ixaf050. 10.1093/isd/ixaf050

[B30] Moradmand M, Schönhofer AL, Jäger P (2014) Molecular phylogeny of the huntsman spider family Sparassidae with focus on the genus *Eusparassus* and notes on the RTA-clade and “Laterigradae”. Molecular Phylogenetics and Evolution 74: 48–65. 10.1016/j.ympev.2014.01.021 24508702

[B31] Pett BL, Ferreira E, Agnarsson I (2025) Megadiverse Madagascar: new species and genera of argyrodine spiders (Araneae, Theridiidae, Argyrodinae) from the eastern rainforest reserve Analamazaotra. Insect Systematics and Diversity 9: ixaf047. 10.1093/isd/ixaf047

[B32] Querner P, Szucsich N, Landsberger B, Erlacher S, Trębicki L, Grabowski M, Brimblecombe P (2022) Identification and spread of the ghost silverfish (*Ctenolepisma calvum*) among museums and homes in Europe. Insects 13(9): 855. 10.3390/insects13090855 PMC9505982 36135556

[B33] Rajaonarimalala R, Korol Y, Andrianarimisa A, Dröge S, Fulgence TR, Grass I, Kreft H, Osen K, Rakotomalala AANA, Rakouth B, Ranarijaona HLT, Randriamanantena R, Ratsoavina FM, Ravaomanarivo LHR, Raveloaritiana E, Schwab D, Soazafy MR, Tscharntke T, Wurz A, Hölscher D, Martin DA (2024) Complex stands in forested tropical landscapes harbor more endemic biodiversity and ecosystem functions. Global Ecology and Conservation 54: e03154. 10.1016/j.gecco.2024.e03154

[B34] Ratnasingham S, Hebert PDN (2007) Bold: The Barcode of Life Data System (http://www.barcodinglife.org). Molecular Ecology Notes 7: 355–364. 10.1111/j.1471-8286.2007.01678.x PMC1890991 18784790

[B35] Ratnasingham S, Hebert PDN (2013) A DNA-based registry for all animal species: the Barcode Index Number (BIN) system. PLOS ONE 8(8): e66213. 10.1371/journal.pone.0066213 PMC3704603 23861743

[B36] Russell-Smith A, Stork NE (1994) Abundance and diversity of spiders from the canopy of tropical rainforest with particular reference to Sulawesi, Indonesia. Journal of Tropical Ecology 10: 545–558. 10.1017/S0266467400008221

[B37] Saitou N, Nei M (1987) The neighbor-joining method: a new method for reconstructing phylogenetic trees. Molecular Biology and Evolution 4(4): 406–425. 10.1093/oxfordjournals.molbev.a040454 3447015

[B38] Shorthouse DP (2010) SimpleMappr, an online tool to produce publication-quality point maps. https://www.simplemappr.net [Accessed: 1 October 2025]

[B39] Silva-Dávila D (2005) Revision of the spider genus *Chrosioderma* Simon (Araneae: Sparassidae). Proceedings of the California Academy of Sciences 56: 337–377.

[B40] Simon E (1880) Révision de la famille des Sparassidae (Arachnides). Actes de la Société Linnéenne de Bordeaux 34(2/3/4): 223–351.

[B41] Simon E (1903) Histoire naturelle des araignées. Deuxième édition, tome second. Roret, Paris, 669–1080.

[B42] Soutinho JG, Couto H, Andreone F, Crottini A (2018) When camouflage fails: Predation of a huntsman spider *Damastes* sp. (Araneae: Sparassidae) on a stick insect *Antongilia* sp. (Phasmatodea: Bacillidae: Antongiliinae) from Madagascar. Acta Arachnologica 67(1): 31–33. 10.2476/asjaa.67.31

[B43] Srivathsan A, Lee L, Katoh K, Hartop E, Kutty SN, Wong J, Yeo D, Meier R (2021) ONTbarcoder and MinION barcodes aid biodiversity discovery and identification by everyone, for everyone. BMC Biology 19: 1–21. 10.1186/s12915-021-01141-x PMC8479912 34587965

[B44] Srivathsan A, Feng V, Suárez D, Emerson B, Meier R (2024) ONTbarcoder 2.0: rapid species discovery and identification with real-time barcoding facilitated by Oxford Nanopore R10.4. Cladistics 40(2): 192–203. 10.1111/cla.12566 38041646

[B45] Tin MMY, Economo EP, Mikheyev AS (2014) Sequencing degraded DNA from non-destructively sampled museum specimens for RAD-tagging and low-coverage shotgun phylogenetics. PLOS ONE 9(5): e96793. 10.1371/journal.pone.0096793 PMC4020769 24828244

[B46] Trębicki Ł, Patoleta BM, Dabert M, Żabka M (2021) Redescription of type species of the genus *Cytaea* Keyserling, 1882 (Araneae: Salticidae)—an integrative approach. The European Zoological Journal 88(1): 933–947. 10.1080/24750263.2021.1961029

[B47] Vieilledent G, Grinand C, Rakotomalala FA, Ranaivosoa R, Rakotoarijaona JR, Allnutt TF, Achard F (2018) Combining global tree cover loss data with historical national forest cover maps to look at six decades of deforestation and forest fragmentation in Madagascar. Biological Conservation 222: 189–197. 10.1016/j.biocon.2018.04.008

[B48] World Spider Catalog (2026) World Spider Catalog. Version 27. Natural History Museum Bern. http://wsc.nmbe.ch, accessed on 10 Mar 2026. 10.24436/2

[B49] Wood HM, Griswold CE (2022) Araneae, Spiders, Foka, Foko, Hala. In: Goodman S (Ed.) The New Natural History of Madagascar. Princeton University Press, Princeton, 878–893. 10.2307/j.ctv2ks6tbb.113

[B50] Wood HM, Scharff N (2018) A review of the Madagascan pelican spiders of the genera *Eriauchenius* O. Pickard-Cambridge, 1881 and *Madagascarchaea* gen. n. (Araneae, Archaeidae). ZooKeys 695: 1–56. 10.3897/zookeys.727.20222 PMC5799789 29416388

[B51] Zhang J, Pan T, Zhang H, Xing Y, Yu H, Zhong Y (2025) Species delimitation of newly collected spiders of the genus *Pseudopoda* (Araneae, Sparassidae) from Honghe Hani and Yi Autonomous Prefecture, Yunnan, China: an integrated morphological and molecular approach. Zoosystematics and Evolution 101(1): 141–171. 10.3897/zse.101.136177

