# Supplementary material for: Redescription of Damastes leo (Simon, 1903), comb. nov. (Araneae, Sparassidae) from Madagascar with first description of the male
Source: Zookeys. 2026 Jun 17;1282:287–303. doi: 10.3897/zookeys.1282.192182 (PMC13294686; doi:10.3897/zookeys.1282.192182)
Supplement: Supplementary material 1 — Data on the distribution of Damastes leo from iNaturalist [file zookeys-1282-287_article-192182__-s001.docx]

**Supplementary material 1**

**table S1.** Data on the distribution of *Damastes leo* from iNaturalist. Coordinates and geographical regions are named and standardized, as well as the sex of the spotted spider. The last check was carried out on 1 October 2025. m **–** male, f **–** female.

| **Coordinates** | **District or Region** | **Locality** | **Sex** | **iNaturalist observation** |
| --- | --- | --- | --- | --- |
| 14°42'48.6"S 49°27'57.0"E | d'Andapa | Anjanaharibe-Sud Special Reserve | f | marojejy 2021 https://www.inaturalist.org/observation/69808893 |
| 15°41'20.8"S 49°59'19.3"E | Analanjirofo | Masoala rainforest | f | Matthias D. Frei https://www.domani.ch/masoala/en/spider.html |
| 18°35'01.6"S 47°58'11.4"E | Vodivato | Anjozorobe-Angavo Protected Area | m | alexlaube 2018 https://www.inaturalist.org/photos/112468228 |
| 18°47'34.7"S 48°25'35.0"E | Alaotra-Mangoro | Mantadia NP | f | danielaustin 2019 https://www.inaturalist.org/photos/54204860 |
| 18°48′12.1″S, 48°25′04.5″E | Alaotra-Mangoro | Mantadia NP | f | davidrabehevitra 2019  https://www.inaturalist.org/photos/31559952 |
| 18°56′06.9″S, 48°25′40.9″E | Alaotra-Mangoro | Analamazaotra NP | f | phiro 2007  https://www.inaturalist.org/observations/149504553 |
| 18°56'12.0"S 48°24'53.8"E | Alaotra-Mangoro | Analamazaotra NP | m | msyygod 2024  https://www.inaturalist.org/photos/524329277 |
| 18°56′12.5″S, 48°24′53.9″E | Alaotra-Mangoro | Analamazaotra NP | m | globalherping 2017  https://www.inaturalist.org/photos/396520918 |
| 18°56′19.2″S, 48°25′05.9″E | Alaotra-Mangoro | Analamazaotra NP | m | irene_vertua 2024  https://www.inaturalist.org/photos/459667706 |
| 18°56'19.8"S 48°26'01.6"E | Alaotra-Mangoro | Analamazaotra NP | f | dimitrikaenel 2019 https://www.inaturalist.org/photos/613602117 |
| 18°56′32.1″S, 48°25′01.5″E | Alaotra-Mangoro | Mitsinjo | m | gauvain_saucy 2024  https://www.inaturalist.org/photos/468604325 |
| 18°56'49.8"S 48°25'09.9"E | Alaotra-Mangoro | Analamazaotra NP | f | finnharrigan 2024 https://www.inaturalist.org/photos/358649855 |
| 19°00'47.5"S 48°27'14.6"E | Alaotra-Mangoro | Iaroka Forest | f | onjalalaina 2019  https://www.inaturalist.org/photos/97815514 |
| 19°00′48.2″S, 48°28′34.6″E | Alaotra-Mangoro | Iaroka Forest | f | martin_ingemansson 2024  https://www.inaturalist.org/photos/463557339 |
| 21°14'27.1"S 47°23'39.6"E | Fianarantsoa | Ranomafana NP | f | ybhagwan 2024 https://www.inaturalist.org/photos/606790059 |
| 21°15'06.0"S 47°24'13.9"E | Fianarantsoa | Ranomafana NP | m+f | dschok 2025  https://www.inaturalist.org/photos/580422709 |
| 21°15'06.5"S 47°24'13.8"E | Fianarantsoa | Ranomafana NP | f | leslieghana 2019 https://www.inaturalist.org/photos/540508596 |
| 21°15′07.4″S, 47°24′55.5″E | Fianarantsoa | Ranomafana NP | m+f | marcopogon 2018  https://www.inaturalist.org/photos/464279035 |
| 21°15′14.1″S, 47°25′17.5″E | Fianarantsoa | Ranomafana NP | f | marcinwiorek 2020 https://www.inaturalist.org/photos/259133481 |
| 21°15′14.7″S, 47°25′19.1″E | Fianarantsoa | Ranomafana NP | f | briangratwicke 2014  https://www.inaturalist. org/photos/1421958 |
| 21°15'15.1"S 47°24'25.6"E | Fianarantsoa | Ranomafana NP | f | carla_vh88 2025 https://www.inaturalist.org/photos/580896396 |
| 21°15′22.6″S, 47°24′35.8″E | Fianarantsoa | Ranomafana NP | f | lemurtaquin 2014  https://www.inaturalist.org/photos/57442632 |
| 21°15′24.0″S, 47°25′14.1″E | Fianarantsoa | Ranomafana NP | f | antoine_baglan 2025  https://www.inaturalist.org/photos/479500730 |
| 21°15′27.7″S, 47°24′54.1″E | Fianarantsoa | Ranomafana NP | f | demianhiss 2017  https://www.inaturalist.org/photos/359525729 |
| 21°15'35.5"S 47°27'05.4"E | Fianarantsoa | Ranomafana NP | m | saemmi 2016 https://www.inaturalist.org/photos/631452737 |
| 21°15'50.6"S 47°25'09.5"E | Fianarantsoa | Ranomafana NP | m | charleyhesse 2000 https://www.inaturalist.org/photos/87570001 |
| 21°30′13.4″S, 47°25′16.06″E | Fianarantsoa | Ikongo | m | bruno_meriguet 2023  https://www.inaturalist.org/photos/331495786 |
| 22°35′37.4″S, 47°41′53.1″E | Fianarantsoa | Angavokely | f | nanciart_mada 2017  https://www.inaturalist.org/photos/102374299 |
